# Enhanced YAP expression leads to EGFR TKI resistance in lung adenocarcinomas

**DOI:** 10.1038/s41598-017-18527-z

**Published:** 2018-01-10

**Authors:** Ting-Fang Lee, Yu-Chi Tseng, Phung Anh Nguyen, Yu-Chuan Li, Chao-Chi Ho, Cheng-Wen Wu

**Affiliations:** 1 Institute of Clinical Medicine, National Yang-Ming University, Taipei, Taiwan; 2 Institute of Biochemistry and Molecular Biology, National Yang-Ming University, Taipei, Taiwan; 3 College of medical Science and Technology, Taipei Medical University, Taipei, Taiwan; 4 Department of Population & Quantitative Health Sciences, School of Medicine, Case Western Reserve University, Ohio, USA; 5 Dermatology Department, Wan-Fang Hospital, Taipei, Taiwan; 6 Department of Internal Medicine, National Taiwan University Hospital and National Taiwan University Medical College, Taipei, Taiwan; 7 Institute of Biomedical Sciences, Academia Sinica, Taipei, Taiwan

## Abstract

Epidermal growth factor receptor (EGFR) mutation is prevalently expressed in lung adenocarcinoma cases and acts as one of the major driving oncogenes. EGFR tyrosine kinase inhibitors (TKIs) have been used in patients with EGFR-mutant as an effective targeted therapy in lung adenocarcinoma, but drug resistance and tumor recurrence inevitably occurs. Recently, Yes-associate protein (YAP) has been reported to promote multiple cancer cell properties, such as promoting cell proliferation, epithelial-mesenchymal transition and drug resistance. This study investigated the roles of YAP in TKI-resistant lung adenocarcinoma. In TKI-sensitive cells, enhanced YAP expression leads to TKI resistant. Also, upregulated YAP expression and activation were detected in long-term TKI-induced resistant cells. With reduced YAP expression using shRNA or YAP inhibitors, TKI-resistant cells become TKI-sensitive. reduced xenograft tumor size in nude mice and Moreover, combined EGFR TKI and a YAP inhibitor, statin, prolonged survival among lung cancer patients analyzed by Taiwan National Health Insurance Research database. These observations revealed the importance of YAP in promoting TKI-resistance and combined YAP inhibition can be a potential therapy delaying the occurrence of TKI-resistance in lung adenocarcinoma.

## Introduction

Epidermal growth factor (EGFR) tyrosine kinase inhibitors (TKIs) are the standard care for patients with EGFR-mutant lung adenocarcinoma. Although patients benefit from the TKI therapies, they acquire drug resistance within approximately 9–14 months. A variety of mechanisms lead to the resistance. A secondary EGFR mutation, T790M, is the most frequent cause of resistance^1^. Besides, oncogene shift or activation of other pathways including amplification of MET or HER2 and activation of KRAS that lead to the activation of downstream survival signaling^2–4^.

Resistance mechanisms, which are poorly understood and vary considerably, are important for the design of novel targeted therapies aimed at preventing resistance. Recently, a next-generation EGFR inhibitor, AZD9291, has been approved to fight against EGFR T790M, the most frequent cause of TKI-resistance^5^. Despite the significant clinical responses to such targeted therapies, cancers are not cured, as most tumors acquire resistance. Due to the wide variety of identified resistance mechanisms, studies that focused on developing strategies to overcome individual resistance mechanisms may encounter further resistance.

An alternative strategy to overcome resistance is to identify a combination treatment approach that inhibits more than one resistance mechanisms. A Hippo signaling effector, YES-associated protein (YAP), has been identified as a key survival input that mediates resistance by acting in parallel to other pathway in tumor progression^6,7^. YAP is a transcriptional coactivator and is important for the regulation of cell growth, tissue homeostasis and organ size control. Upregulated YAP is reported to support cell survival upon the suppression of main driver genes^8,9^.

In this study, we demonstrated upregulated YAP activity in TKI-resistant cells and thus, we investigated the effectiveness of cotargeting EGFR and YAP using both *in vitro* and *in vivo* methods, and we also analyzed the health insurance database to consolidate the findings. We suggest that combined inhibition of EGFR and YAP can be a promising therapeutic strategy for EGFR mutant lung adenocarcinoma patients.

## Results

### Enhanced YAP expression prevents TKI-induced cytotoxicity

**Figure 1:**
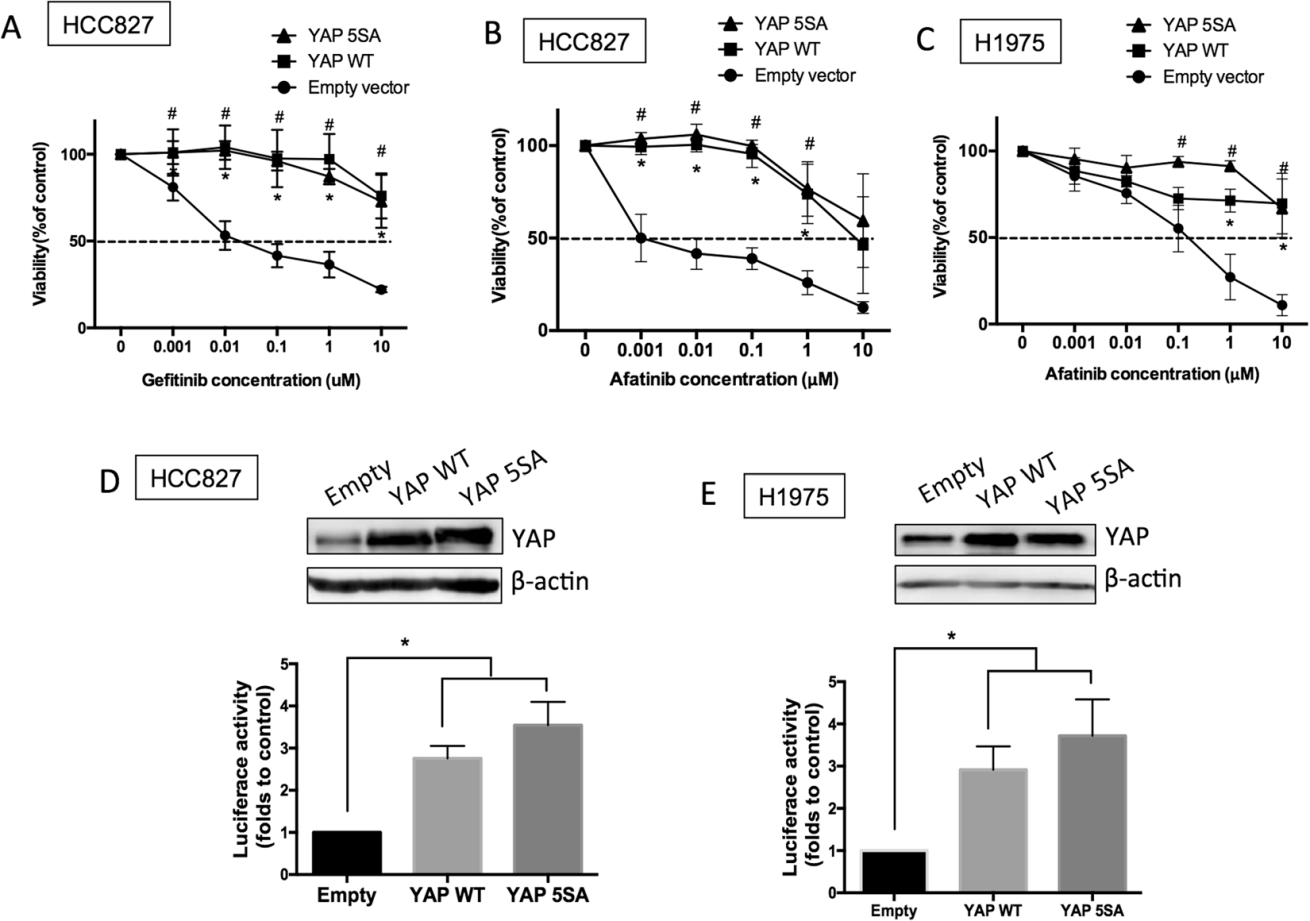
Enhanced YAP expression and activity promotes drug resistance. HCC827 cells overexpressed with wild-type (YAP WT) or constitutively active YAP (YAP 5SA) were cultured in the presence of (**A**) gefitinib or (**B**) afatinib. (**C**) H1975 cells with YAP expression were treated with afatinib. The error bars represent the S.E. of 3 independent experiments. *P < 0.05, empty compared with YAP 5SA, and ^#^P < 0.05, empty compared with YAP WT. Upregulated YAP protein levels and activity were detected in both YAP overexpressed groups in (**D**) HCC827 and (**E**) H1975 cells. The original blot of these samples was shown in Supplementary Figure S5A. The error bars represent the S.E. of 3 independent experiments. *P < 0.05, empty compared with YAP 5SA or YAP WT.

YAP is known to provide a strong survival signal for lung cancer cells. Here, we tested if upregulated YAP expression can maintain EGFR-dependent cells survive in the presence of EGFR TKI. Results showed that, forced YAP expressed HCC827 cells became resistant to EGFR TKIs, both gefitinib and afatinib (Fig. 1A and B). On the other hand, H1975 cells that were sensitive to afatinib became resistant with over expressed YAP (Fig. 1C). Upregulated YAP protein expression and activity were detected in YAP overexpressed groups (Fig. 1D and E). The wild-type YAP and constitutively active YAP (5SA) showed similar effects on the protein expression as well as the activity of YAP. Forced YAP expression rescued cells from TKI treatment gave us an idea that enhanced YAP expression may lead to TKI resistance.

To verify this hypothesis, cell lines resistant to either first- or second-generation TKIs were developed in HCC827 or H1975 cells using gradual increasing subtoxic doses of TKI treatment (Table S1). Compared to their parental TKI-sensitive lines, the resistant lines, HCC827^AR^, HCC827^GR^ and H1975^AR^, showed significantly increased cell viability in the presence of TKI treatment (Figure S1A–C). Secondary EGFR T790M mutation was not detected in either HCC827^AR^ or HCC827^GR^ cells using PCR method (data not shown).

**Figure 2:**
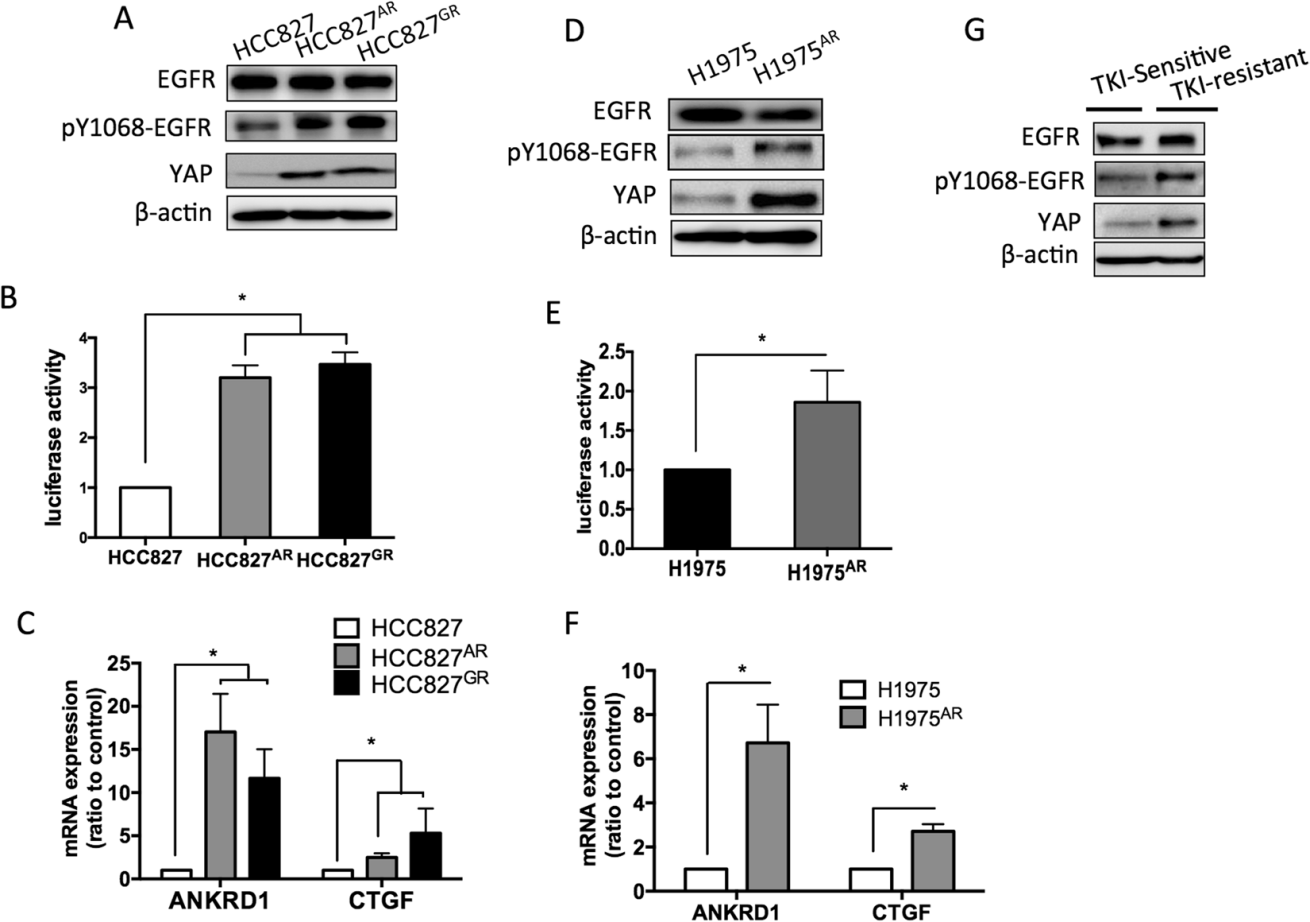
Enhanced YAP expression and activity in TKI-resistance cells. Compared to the parental HCC827 cells, the two resistant lines, HCC827^AR^ and HCC827^GR^ cells showed enhanced (**A**) YAP protein levels, (**B**) promoter activity and (**C**) ANKRD1 and CTGF mRNA levels. Upregulated YAP expression and activity were also found in H1975^AR^ cells compared to H1975 cells (**D**, **E** and **F**). The error bars represent the S.E. of 3 independent experiments. *P < 0.05, parental compared with resistant lines. (**G**) Primary cells collected from plural effusion. Cells collected from TKI-resistant patient showed upregulated YAP level. The original blots were shown in Supplementary Figure S5B.

To answer the question whether upregulated YAP caused drug resistance, the protein levels and activities of YAP were detected. In HCC827 group, two TKI-resistant cell lines HCC827^AR^ or HCC827^GR^ showed enhanced YAP protein expression (Fig. 2A) together with enhanced YAP activity detected by 8XGTIIC reporter assay (Fig. 2B). Upregulated expression of target genes, *ANKRD1* and *CTGF* were also detected in the resistant lines (Fig. 2C). In the H1975 group, enhanced YAP expression and activity was detected in H1975^AR^ cells (Fig. 2D–F). Upregulated YAP expression was also detected in the primary lung cancer cells from patients acquired TKI-resistance (Fig. 2G). Interestingly, among these 3 groups, the phospho-EGFR levels in the resistant lines were higher compared to that of the TKI sensitive cells (Fig. 2A,D and G).

The upregulation of YAP in TKI-resistant cells inspired us to explore the possible driver genes that promoted YAP in the resistant lines. To reduce the complexity of the heterogeneous resistant population, single clonal lines were isolated: five HCC827^GR^, three HCC827^AR^ and five H1975^AR^ clones. Interestingly, all the lines showed high YAP levels compared to the parental heterogeneous lines (Figure S2A,B and C). c-Met or Her2 amplification has been reported to be a cause of drug resistance. Here we detect an up regulation of Her2 mRNA expression in many of the resistant clonal lines, suggesting a possible role of Her2 in the TKI-resistance. As we knocked down Her2 with two different shRNAs, reduced YAP expression was detected (Figure S2G).

### Sustained YAP expression is essential for the survival of TKI-resistant cells

**Figure 3:**
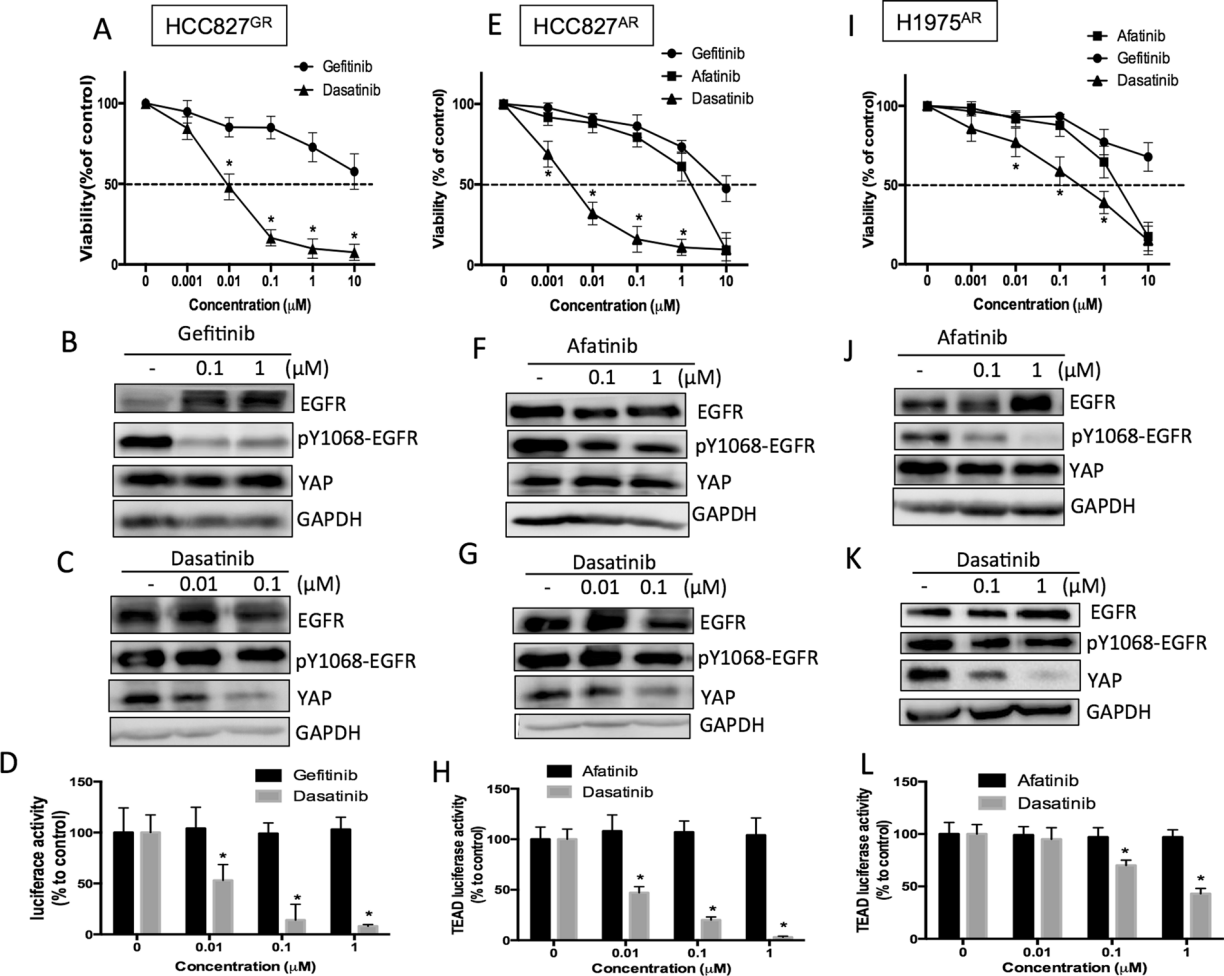
TKI that reduced YAP levels caused cytotoxicity in the resistant lines. Cell viability assays performed in three resistant lines: (**A**) HCC827^GR^ (**B**) HCC827^AR^ or (**C**) HCC827^GR^ cells in the presence of gefitinib afatinib or dasatinib. Immunoblotting demonstrated YAP protein levels (**B**, **C**,**F**,**G**,**J** and **K**) or activity (**D**,**H** and **L**) in the presence of different TKIs in resistant lines. The original blots were shown in Supplementary Figure S6.

Knowing that YAP is an important survival factor for TKI-resistant cells, we next explored whether sustained YAP level is a key factor for the survival of TKI-resistance. Gefitinib, had no significant cytotoxicity on HCC827^GR^ cells (Fig. 3A), showed no effect on YAP protein level and activity (Fig. 3B and D). A src family kinase inhibitor, dasatinib, known to reduce YAP expression, on the other hand, reduced the viability of HCC827^GR^ cells (Fig. 3A) with reduced YAP protein levels and activity (Fig. 3C and D). The two afatinib resistant lines, HCC827^AR^ and H1975^AR^ were not sensitive to the treatment of afatinib or gefitinib (Fig. 3E and I) had no significant effect on YAP protein levels and activity (Fig. 3F,H,J and L); while dasatinib significantly caused cytotoxicity (Fig. 3E and I) and reduced YAP levels and activity (Fig. 3G,H,K and L). Interestingly, gefitinib and afatinib, the two EGFR TKIs, though significantly inhibited EGFR phosphoryation, had no effect on YAP levels and cell viability. On the other hand, dasatinib, that had no significant effect on EGFR phosphorylation, reduced YAP levels and more importantly, cell viability. Theses results suggested the importance of YAP level on the survival of TKI-resistant cells.

### Combined EGFR and YAP inhibition effectively reduced the viability of TKI-resistant cells

**Figure 4:**
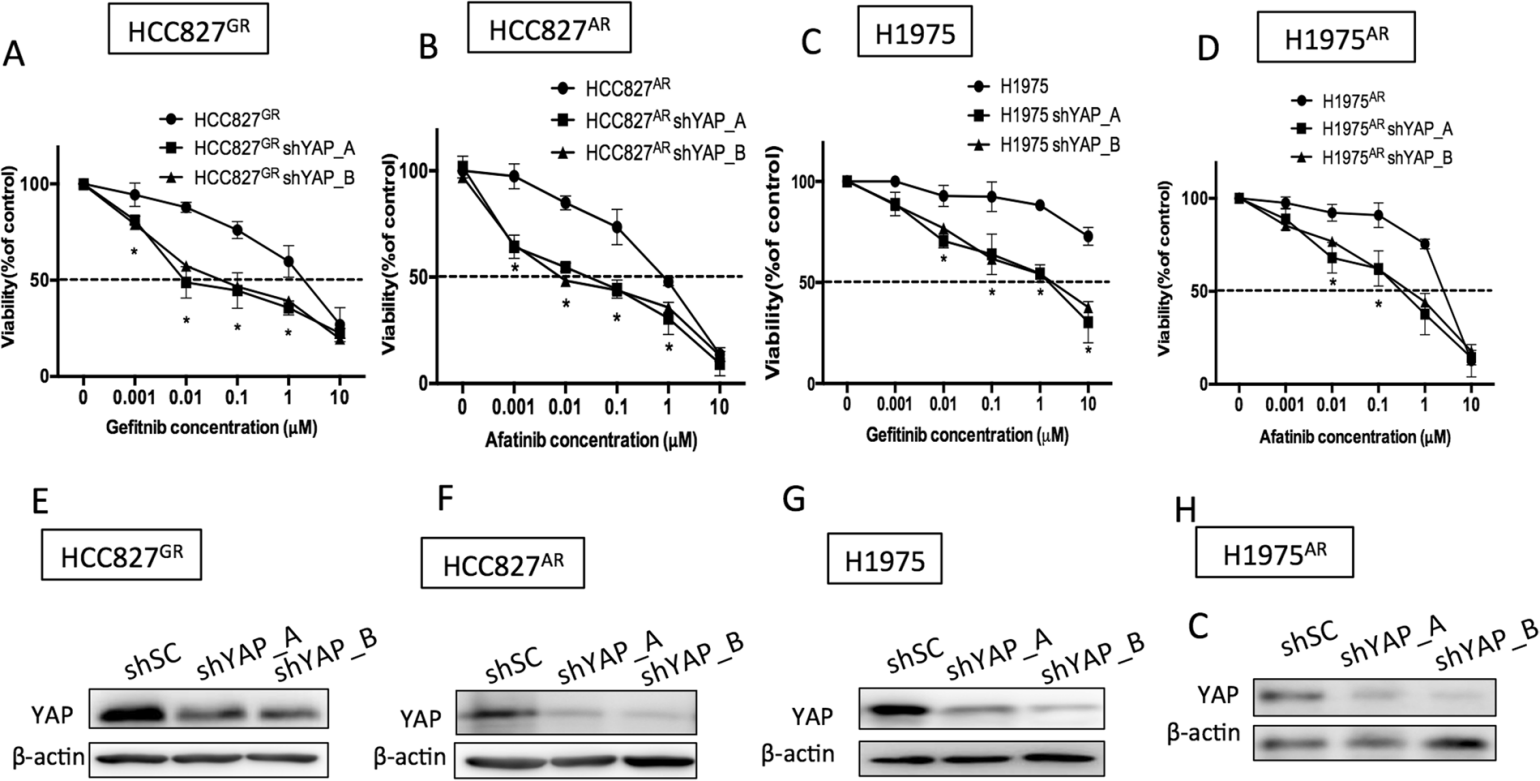
Reduce YAP expression by shRNAs re-sensitized the resistant lines. Gefitinib or afatinib-induced cytotoxicity was performed in 4 TKI-resistant cell lines: (**A**) HCC827^GR^, (**B**) HCC827^AR^ (**C**) H1975 or (**D**) H1975^AR^ in the presence of scramble or YAP shRNAs. The error bars represent the S.E. of 3 independent experiments. *P < 0.05, shSC compared with YAP knockdowns. Reduced YAP expression was detected in the YAP knockdown grouped (**E–H**). The original blots were shown in Supplementary Figure S7A.

**Figure 5:**
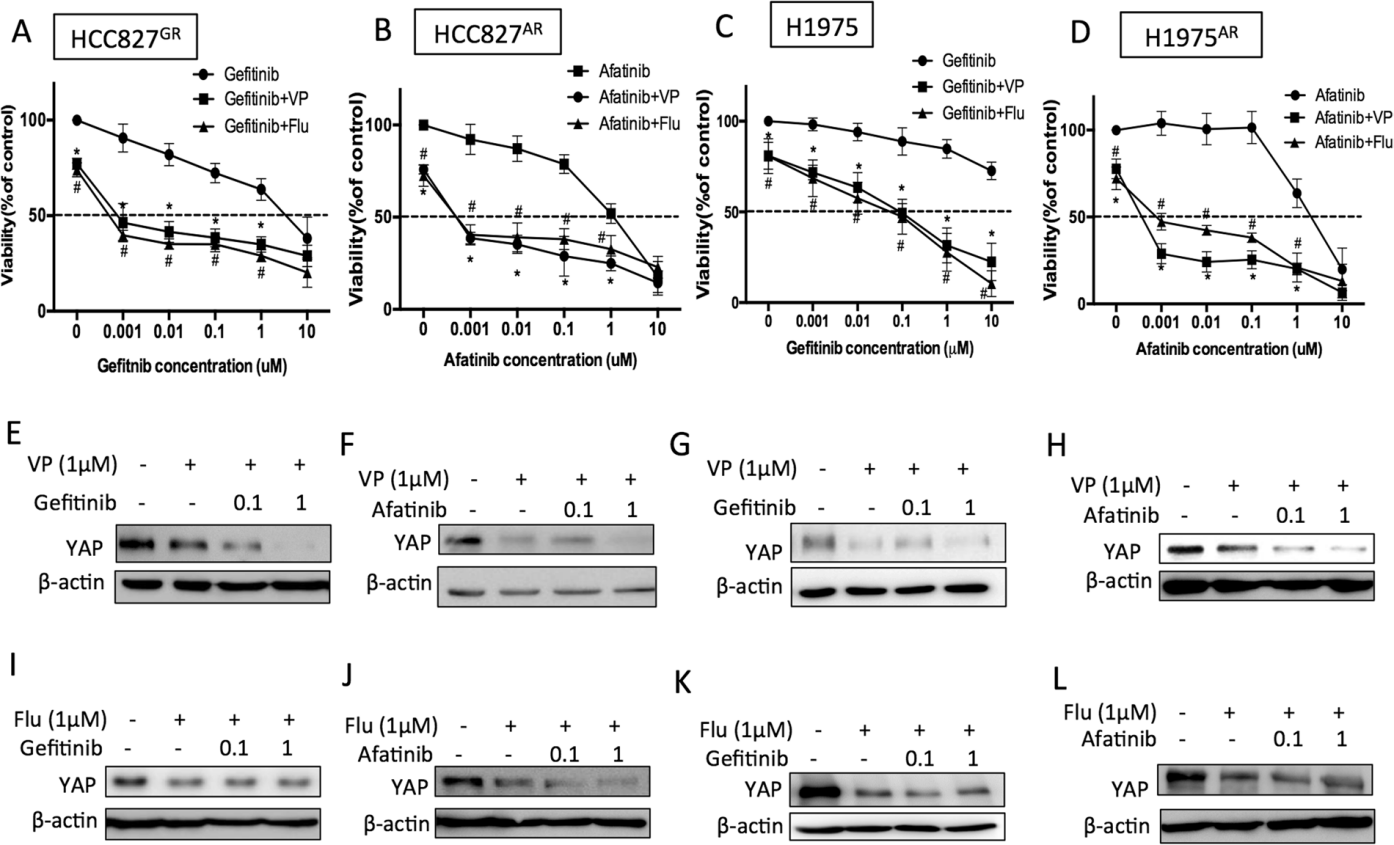
Pharmaceutical suppression of YAP re-sensitized the resistant lines. Gefitinib or afatinib-induced cytotoxicity was performed in 4 TKI-resistant cell lines: (**A**) HCC827^GR^, (**B**) HCC827^AR^ (**C**) H1975 or (**D**) H1975^AR^ in the presence of vehicle control (0.01% DMSO), verteporfin (VP; 1 μM) or fluvastatin (Flu; 1 μM). The error bars represent the S.E. of 3 independent experiments. *P < 0.05, vehicle control compared with VP, and ^#^P < 0.05, vehicle compared with Flu. TKI-resistant cells cultured in the presence or absence of gefitinib or afatinib combined with verteporfin (**E–H**) or fluvastatin **(I-L**) for 48 h. Immunoblotting demonstrated YAP levels. The original blots were shown in Supplementary Figure S7B and C.

Because the TKI resistant lines showed high phospho-EGFR levels (Fig. 2A,D and G) and because of the crucial roles of YAP in supporting tumor survival in the presence of TKIs, we next aimed to identify whether YAP is an ideal target for combined inhibition with EGFR TKI. First, we reduced YAP levels using two different YAP shRNAs. Results showed that, YAP knockdowns were more sensitive to the treatment of TKIs compared to the control groups and this effect was demonstrated in the four TKI resistant lines (HCC827^GR^, HCC827^AR^, H1975 or H1975^AR^) (Fig. 4A–D). Immunoblotting showed reduced YAP expression with the two YAP shRNAs (Fig. 4E–H). Moreover, reduced cell proliferation was detected in YAP knockdowns (Figure S3A–C). Also, pharmaceutical inhibition of YAP using either verteporfin or fluvastatin (Fig. 5A–D) effectively turned the TKI-resistant cells into TKI-sensitive. Reduced YAP expressions in the presence of verteporfin (Figure S3D–F) or fluvastatin (Figure S3G–I) were detected by immunoblotting. Reduced YAP expressions were also detected in the presence of gefitinib or afatinib combined with verteporfin (Fig. 5E–H) or fluvastatin (Fig. 5I–L). These results suggest that combined EGFR and YAP inhibition effectively reduced the viability of TKI-resistant cells.

**Figure 6:**
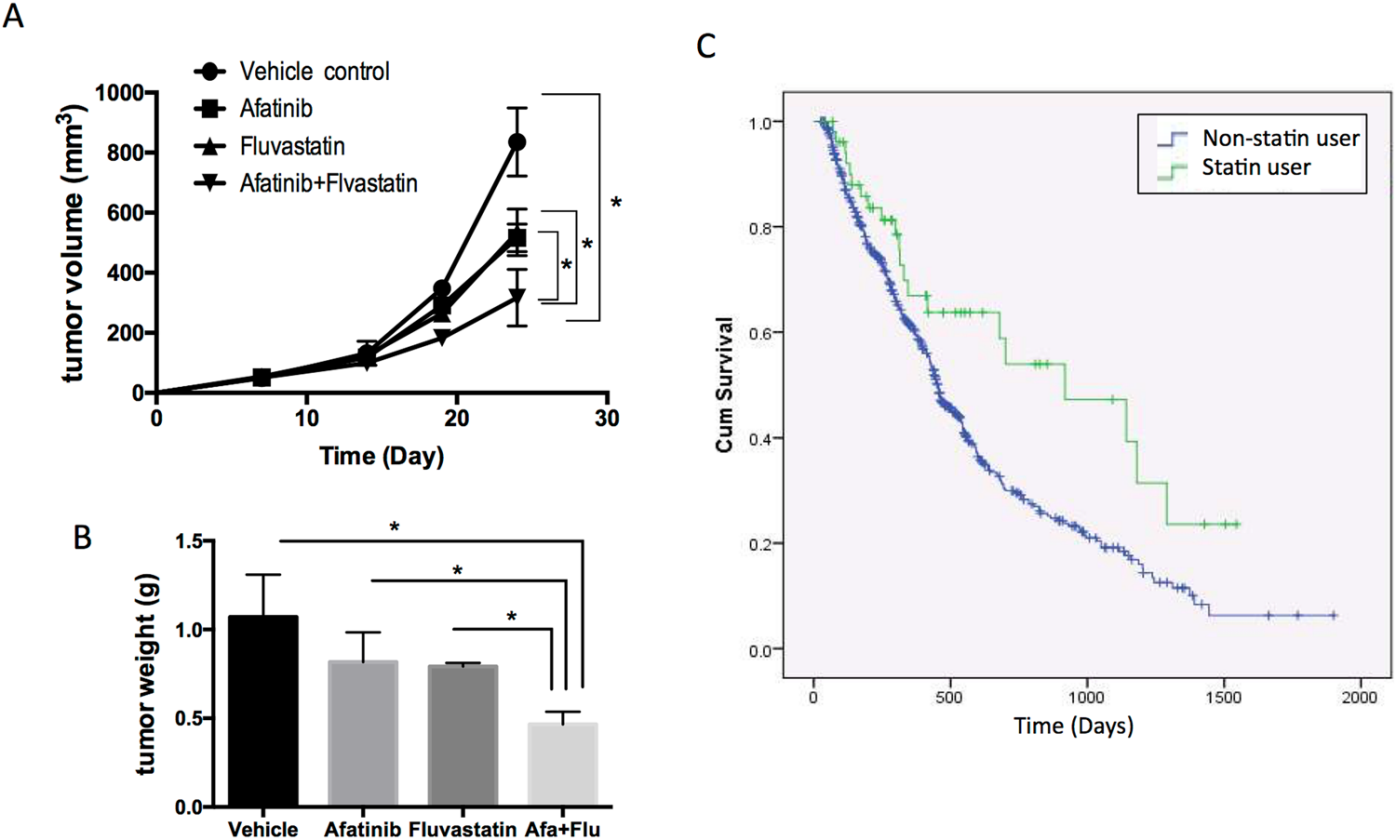
Effects of combined therapy of TKI and statin, as a YAP inhibitor. Subcutaneous xenograft model demonstrated the effect of combined therapy of afatinib and fluvastatin in the inhibition of TKI-resistant tumor. (**A**) Tumor size and (**B**) tumor weight measured in either vehicle or drug treated groups. (**C**) Analysis of the Taiwan National Health Insurance data sources revealed prolonged survival in TKI prescribed patients with regular statin use compared to no statin used patients.

Moreover, a subcutaneous xenograft model was used to further demonstrate the effectiveness of combined therapy of TKI and YAP inhibitor, statin. We injected HCC827^AR^ cells into nude mice. A week after inoculation, vehicle, afatinib or fluvastatin was administered to mice. Compared to either vehicle or single drug administered groups, combined therapy significantly suppressed tumor growth (Figs 6A and S4) and tumor weight (Fig. 6B).

### Better survival in lung cancer patients with TKI and statin

To gain more clinical insights of the effects of combined YAP and EGFR inhibition in lung cancer, we analyzed the effects of statin use in lung cancer patients with EGFR TKI prescription using Taiwan National Health Insurance Research Database. We collected the data from all the TKI prescribed lung cancer subjects. Among them, subjects who happened to have statin prescription, probably due to hyperlipidemia, were grouped as statin user. Precisely, the study cohort comprised 2,392 patients diagnosed with lung cancer from 2007 to 2012. Among the 2,392 lung cancer patients, 594 identified with cumulative TKI use for more than 30 days. Out of the 594 patients, 53 had statin prescribed for more than 30 days after the diagnosis of lung cancer. Kaplan–Meier and Cox proportional regression model were used with adjusting for age and sex. Comparing to the group of TKI used patients, patients had statin prescription plus TKI had better survival results (Kaplan-Meier log-rank test, p-value = 0.007) (Fig. 6C).

## Discussion

This study demonstrated that enhanced YAP expression promoted the resistance of EGFR TKI in lung adenocarcinoma cells. In consistent with this finding, an upregulated YAP expression was shown in TKI-resistant cells. Using combination approach, combined EGFR and YAP inhibition significantly increased the efficacy of TKIs. Health insurance database also confirmed the beneficial effects of the combined therapy of TKI and statin, which acts as a YAP inhibitor. This study suggests that cotargeting EGFR and YAP is an effective strategy in EGFR mutant lung cancer.

Resistance mechanisms, which are poorly understood and vary considerably, are important for designing new targeted therapies. A secondary EGFR mutation, T790M, is the most frequent cause of resistance. Next-generation EGFR inhibitors were developed to fight against this most frequent cause of TKI-resistance. Although AZD9291 has recently been approved to target EGFR T790M, acquired resistance with EGFR C797S been reported^10^. Due to the property of genome instability of cancer cells, a cotargeting strategy may work as an alternative to delay further resistance. Here, we demonstrated that combined inhibition of EGFR and YAP significantly increased the susceptibility of TKI-resistant cells, both EGFR T790M-containing and non-T790M-containing cells. These results suggest that YAP works as a key survival input that mediates resistance in parallel to EGFR pathway.

Due to the complexity of TKI resistance, a variety of TKI-resistant lines were used in this study and each of them represents different subgroup of the complicated resistance mechanisms. HCC827 cells prone to show c-Met amplification during the development of resistance^2^ due to the pre-existence of c-Met in a small group of cells that leads to the clonal selection effect^11^. However, c-Met amplification was not detected in our HCC827 resistant lines, neither in HCC827^GR^ nor in HCC827^AR^. Instead, Her2 amplification was detected in some of those clones, suggesting the heterogeneity of HCC827 cells. On the other hand, H1975 cells, though generated before the use of TKI, harbor EGFR T790M and have been widely used as a TKI-resistant line *in vitro*. The second generation TKI afatinib though showed strong efficacy on the T790M-containing cells *in vitro*, its response rate in patients with T790M is not satisfactory^12^. The H1975^AR^ we generated *in vitro*, can represent a small subgroup of T790M-containing cell that gained further TKI-resistant ability.

YAP has been identified as a key determinant to enhance treatment sensitivity in EGFR-targeted therapies in lung cancer^13,14^. Apart from the T790M-dependent H1975 and T790M-independent HCC827 cells used in previous studies^13,14^, here we also developed TKI-resistant lines and demonstrated the effects of YAP inhibition in those resistant lines. Upregulation of YAP in TKI-induced drug-resistant cell was a novel finding in this study. Due to the difficulty of collecting tissue samples from patients acquired drug resistance, we measured the YAP levels in TKI-induced resistant lines and also primary tumor cells collected from plural effusion. The finding of upregulated YAP in TKI-resistant cells provides us a reason of the use of YAP inhibition in TKI-resistant tumor.

Despite the significant efficacy of dasatinib on TKI-resistant lines demonstrated in this study, a phase II clinical trial didn’t warrant dasatinib as first-line, single-agent therapy in advanced NSCLC^15^. Independent phase I/II clinical trials also demonstrated unsatisfactory results of combined dasatinib and gefitinib or elotinib treatment^16–18^. The non-selection of patient population can be a reason for the fail of these clinical trials.

A potential application for this finding is to add an YAP inhibitor that work together with EGFR TKI as a combination therapy. The addition of YAP inhibitor can enhance the efficacy of TKIs. Through the use of *in vitro* assays, we demonstrated that YAP inhibition plus TKI is significantly better than TKI alone at reducing tumor cell viability in both T790M-dependent and T790M-independent cells. The health insurance database analysis also showed a beneficial effect of statin use in TKI prescribed patients, suggesting a synergistic effect of statin and TKIs.

## Materials and Methods

### Cells and Cell culture

The following human lung cancer cell lines were used in this study: H1975 (EGFR L858R and T790M mutations) and HCC827 (EGFR exon 19 deletion). Human embryonic kidney cells HEK 293 T were used to produce viruses for knocking down genes using shRNAs. H1975, HCC827 and HEK293T cells were obtained from the American Type Culture Collection during 2012. Lung cancer cells with gefitinib resistance, HCC827^GR^, or afatinib resistance, HCC827^AR^ and H1975^AR^, were developed by treating cells with sub-toxic doses and gradually increasing the concentration over 60 days to induce drug resistance (Table S1). CLH21, a primary culture of tumor cells from a lung adenocarcinoma patient with EGFR-TKI resistant due to secondary EGFR T790M mutation was harvested from malignant pleural effusion of a patient who was refractory to EGFR-TKI at the National Taiwan University Hospital (NTUH). The lung cancer cells were grown in RPMI-1640 with 10% fetal bovine serum and cultured at 37 °C in a humidified incubator.

### Plasmids

The YAP-responsive synthetic promoter driving the luciferase plasmid 8xGTIIC-Luc (Addgene 34615) and the constitutively active YAP (YAP 5SA; Addgene 27371) plasmids were obtained from Addgene. The full length clone of wild-type YAP was obtained from from the MGC platform supported by Genomic Research Center, National Yang-Ming University.

### Reporter Assay

For the characterization of TEAD activity in lung cancer cells, the cells were transfected the 8xGTIIC-Luc (addgene, #34615) and PRL-TK plasmids. The cells were plated in 6-well plates, and the day after transfection, gefitinib, afatinib or dasatinib was treated for 2 days. Luciferase luminescence was measured using the Dual-Glo luciferase assay kit (Promega, Madison, WI, USA).

### Viability assay

Cells (3 × 10^3^/well) seeded in 96-well plates were treated with different concentrations of gefitinib, afatinib, dasatinib or verteporfin or fluvastatin for 72 h. Cell viability was detected using the MTT reagent, and absorbance at 540 nm was measured.

### ***In vivo*** study of tumor xenograft

Six-week-old male BALB/c mice, weighing 18–20 g, were maintained under specific-pathogen-free conditions. A total of 2 × 10^6^ HCC827^AR^ cells in PBS mixed with an equal amount of Matrigel were injected subcutaneously into both flanks of nude mice. A week after inoculation, mice were randomized into four groups (n = 6 per group): vehicle control; afatinib (12.5 mg/kg); fluvastatin (20 mg/kg); afatinib and fluvastatin. Afatinib or fluvastatin was prepared by dissolving in 1% methylcellulose before administration. Either drug or vehicle was administered to mouse by oral gavage in a schedule of 6 days on plus one day off for two weeks. All animal experiments were performed in accordance with the animal guidelines of the Acdemia Sinica Institute Animal Care and approved by the Animal Care Committee.

### Taiwan National Health Insurance (NHI) data sources and study design

In the observational study, we collected the data of randomly 1 million residents from Taiwan’s NHI claims data, which contains all medical records in between 1998 to 2011. We identified all subjects who newly diagnosed lung cancer (ICD9-CM code 162) from 2000 to 2010. The diagnostic accuracy of lung cancer was confirmed by both specific ICD-9-CM codes and inclusion in the Registry for Catastrophic Illness Patient Database. All subjects were followed until they were censored being lost to follow-up, termination of insurance, death, or end of 2011. Medication information was retrieved from the pharmacy prescription database. Statin drugs were classified as HMG CoA reductase inhibitors (C10AA 01 to C10AA08). Statin medication exposure was analyzed only before the diagnosis of cancer. Patients who had statin prescriptions for at least 60 days in the two-year duration before cancer diagnosis were classified as “statin users”. Patients in the “non-user” group had no or less than two months statin drugs prescriptions.

## Electronic supplementary material

Supplementary Information
