# Supplementary material for: Enhanced YAP expression leads to EGFR TKI resistance in lung adenocarcinomas
Source: Sci Rep. 2018 Jan 10;8:271. doi: 10.1038/s41598-017-18527-z (PMC5762715; doi:10.1038/s41598-017-18527-z)

Enhanced YAP expression leads to EGFR TKI resistance in lung adenocarcinomas

Ting-Fang Lee, Yu-Chi Tseng, Phung Anh Nguyen, Yu-Chuan Li, Chao-Chi Ho, Cheng-Wen Wu

Supplementary information

Material and Methods

Chemicals

Verteporfin and fluvastatin were purchased from Sigma (St. Louis, MO, USA). Afatinib, gefitinib and dasatinib (Selleckchem, Houston, TX, USA) 0.001 to 10 µM were used to investigate cell viability. Verteporfin, fluvastatin, afatinib, gefitinib or dasatinib were all dissolved in dimethyl sulfoxide (DMSO) with a final concentration of DMSO < 0.1 %. The vehicle control group contained 0.1 % DMSO.

shRNA expression

Lentivirus containing short hairpin RNAs (shRNAs) expressed in a lentiviral vector (pLKO. 1-puro) were generated in 293 T cells. Various pLKO plasmids to knockdown YAP, and scrambled control were provided by National RNAi Core Facility of Academia Sinica, Taipei, Taiwan. For lentivirus production, 293T cells in 6-cm dish were transfected with 2.5 µg pLKO.1-puro lentiviral vectors expressing different shRNAs along with 0.25 µg of envelope plasmid pVSVg and 2.5 µg of packaging plasmid pCMVΔR8.91. Virus was collected 48 h after transfection. To prepare various knockdown cells, HCC827^{GR}, HCC827^{AR}, H1975, or H1975^{AR} cells were infected with lentivirus for 24 h, and followed with 2 µg/ml puromycin selection. The sequences of the lentiviral YAP shRNAs were CAGGTGATACTATCAACCAAA and CCCAGTTAAATGTTCACCAAT.

Immunoblotting

Cells were lysed using RIPA buffer with the addition of proteases and phosphatases inhibitors (Roche, Indianapolis, IN, USA). Quantified lysates were separated on an SDS polyacrylamide gel. The blots were probed with the indicated antibodies as follows: EGFR, phospho-EGFR (Y1068), YAP (all from Cell Signaling, Beverly, MA, USA); and actin, GAPDH (Sigma).

Quantitative RT-PCR

RNA was harvested from cells using Trizol. Complementary DNA was prepared using Transcriptor Reverse Transcriptase (Roche). Quantitative PCR was performed using SYBR Green (Applied Biosystems, Foster City, CA, USA). The primers used are listed in Table S2.

Statistical analyses

The data were analyzed using Student's t test or one-way ANOVA. P values <0.05 were considered significant.

Supplementary figures

Figure S1. Development of TKI-resistant lines

HCC827 cells long-termly exposed to sub-toxic doses of gefitinib or afatinib. Compared to the parental HCC827 cells, the resistant lines (A)HCC827^{GR} and (B) HCC827^{AR} were not sensitive to the treatment of gefitinib or afatinib, respectively. (C) H1975^{AR} cells were more resistant to the treatment of afatinib compared to H1975 cells.

Figure S2. Single cell clones isolated from TKI-resistant lines

Protein expression of YAP from single cell clones isolated from TKI-resistant lines (A)cHCC827^{GR}, (B) HCC827^{AR} or (C) H1975^{AR} cells. Moreover, the mRNA levels of EGFR, Met or HER2 were detected in the single clones from (A) HCC827^{GR}, (B) HCC827^{AR} or (C) H1975^{AR} cells.

Figure S3. Reduced YAP expression in the presence of YAP inhibitors

Reduced cell proliferation was detected in the YAP knockdowns (A-C). Reduced YAP expression detected in the presence of verteporfin (D-F) or fluvastatin (G-I).

Figure S4. Reduced tumor size in combined therapy group

Xenograft tumors in vehicle, afatinib, fluvastatin, and co-treated groups.

Figure S5. Original blots displayed in Figures 1 and 2

Original blots of (A) figures 1 D & E and (B) figures 2 A, D & G.

Figure S6. Original blots displayed in Figure 3

Original blots of (A) figures 3 B, F & J and (B) figures 3 C, G & K.

Figure S7. Original blots displayed in Figures 4 and 5

Original blots of (A) figures 4E-H, (B) figures 5E-H and (C) figures 5I-L.

Table S1. Culture conditions for developing TKI resistant cell lines

TKI resistant clones were developed by treating EGFR active mutant cell lines, H1975 or HCC827, with subtoxic doses of TKI for totally 60 days.

Cell line	Drug treated	Conc (μM)/duration (day)	Developed line
H1975	Afatinib	0.1/15 → 0.5/20 → 1.0/25	$H1975^{AR}$
HCC827	Gefitinib	0.2/10 → 2/10 → 5/10 → 10/30	$HCC827^{GR}$
HCC827	Afatinib	0.01/10 → 0.1/20 → 0.5/30	$HCC827^{AR}$

Table S2. Sequences of QPCR primers

Gene	Forward	Reverse
**GAPDH**	GCATTGCCCTCAACGAC	GTCTCTCTCTTCCTCTTGTGC
**YAP**	CTCCCCAGTGACGAGAGAGC	CTAGGTCTGCGACCTCGAC
**CTGF**	CCTGGTCCAGACCACAGAGT	TGGAGATTTTGGGAGTACGG
**ANKRD1**	AGAACTGTGCTGGGAAGACG	GCCATGCCTTCAAAATGCCA
**EGFR**	ACTCATGCTCTACAACCC	CCAATACCTATTCCGTTACAC
**MET**	CCATCCAGTGTCTCCAGAAGTGAT	ATGGTCAGCCTTGTCCCTC
**HER2**	CCATAACACCCACCTCTGCT	ACTGGCTGCAGTTGACACAC

Fig S1

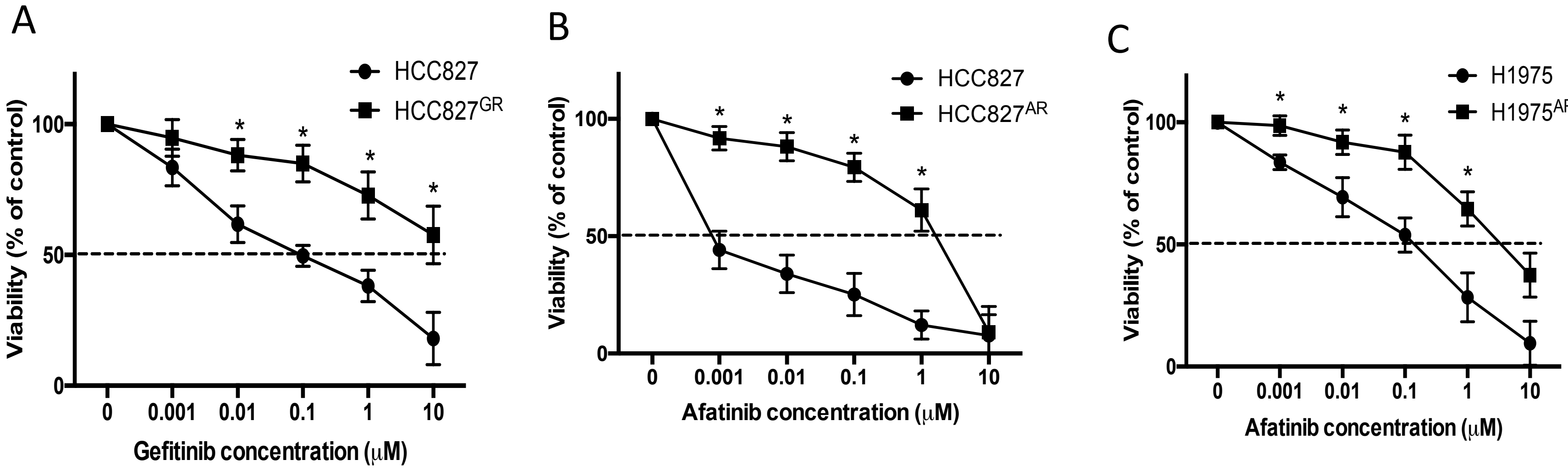
A
HCC827
HCC827GR
Viability (% of control)
100
50
0
0
0.001
0.01
0.1
1
10
Gefitinib concentration (μM)
B
HCC827
HCC827AR
Viability (% of control)
Afatinib concentration (μM)
C
H1975
H1975AR
Viability (% of control)
Afatinib concentration (μM)

Fig S2

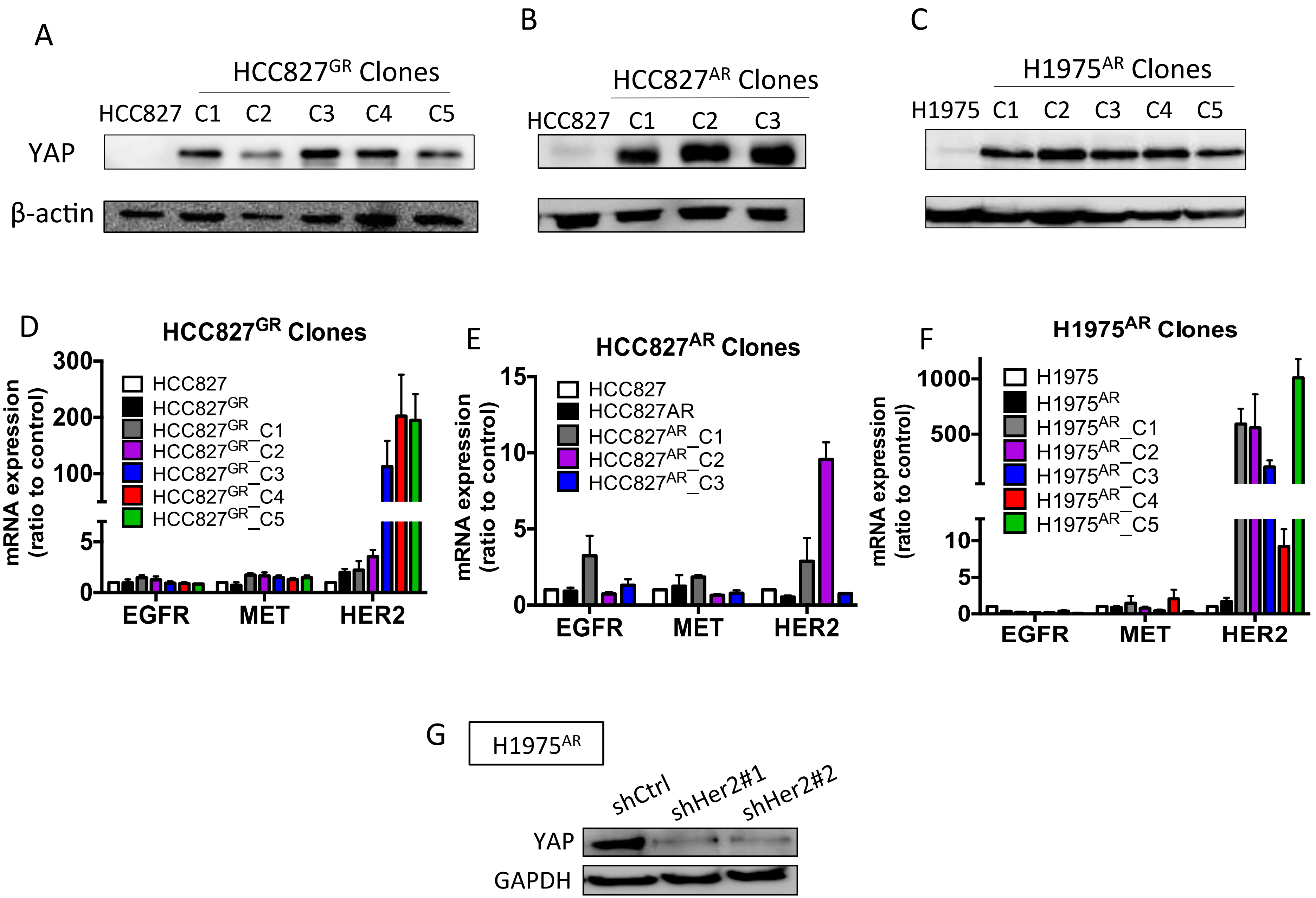
A
HCC827GR Clones
HCC827 C1 C2 C3 C4 C5
YAP
β-actin
B
HCC827AR Clones
HCC827 C1 C2 C3
C
H1975AR Clones
H1975 C1 C2 C3 C4 C5
D
HCC827GR Clones
mRNA expression (ratio to control)
300 200 100 5 0
HCC827
HCC827GR
HCC827GR_C1
HCC827GR_C2
HCC827GR_C3
HCC827GR_C4
HCC827GR_C5
EGFR MET HER2
E
HCC827AR Clones
mRNA expression (ratio to control)
15 10 5 0
HCC827
HCC827AR
HCC827AR_C1
HCC827AR_C2
HCC827AR_C3
EGFR MET HER2
F
H1975AR Clones
mRNA expression (ratio to control)
1000 500 10 5 0
H1975
H1975AR
H1975AR_C1
H1975AR_C2
H1975AR_C3
H1975AR_C4
H1975AR_C5
EGFR MET HER2
G
H1975AR
shCtrl shHer2#1 shHer2#2
YAP
GAPDH

Fig S3

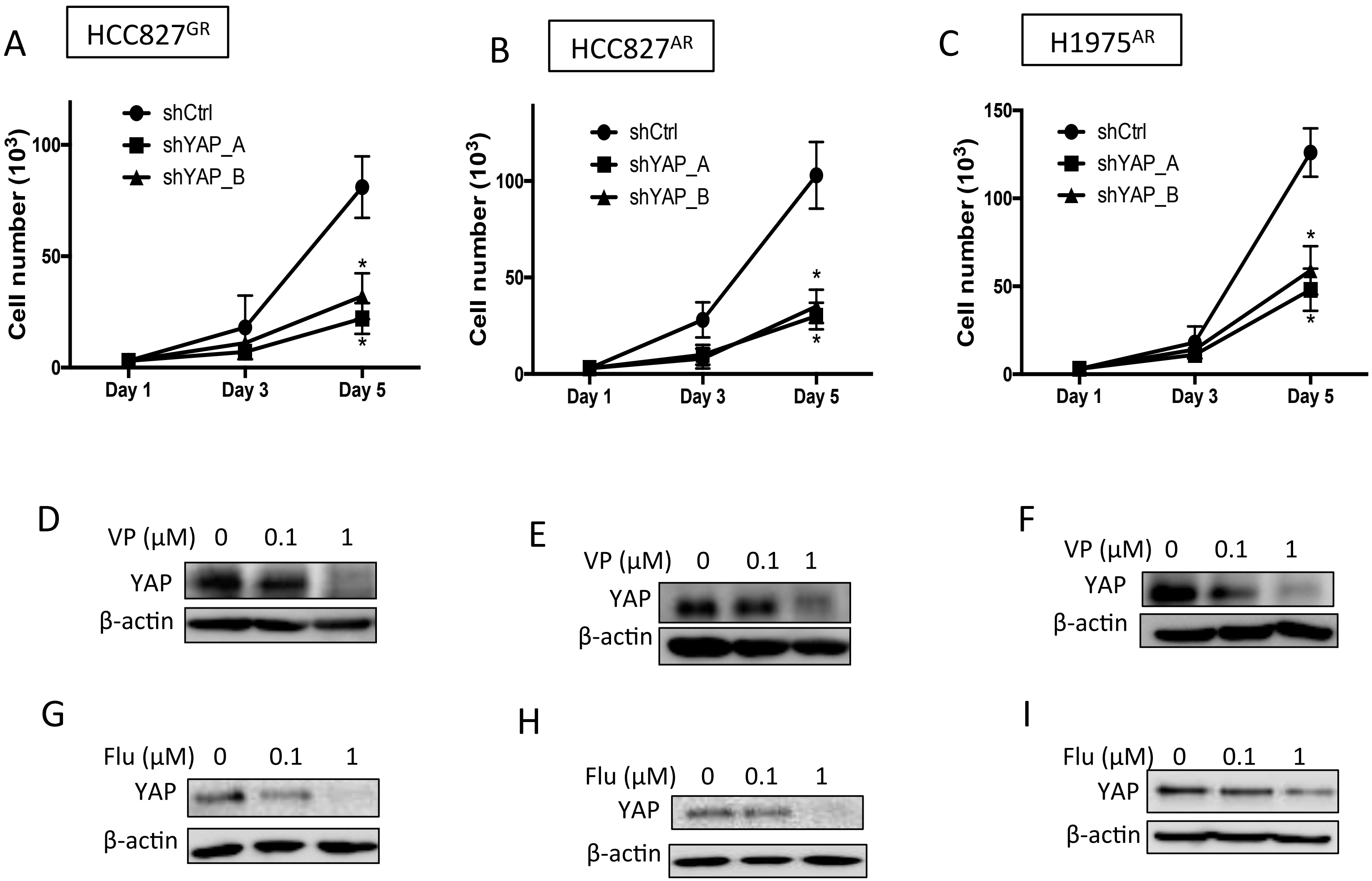
A
HCC827^GR
shCtrl
shYAP_A
shYAP_B
Cell number (10³)
Day 1
Day 3
Day 5
B
HCC827^AR
C
H1975^AR
D
VP (μM) 0 0.1 1
YAP
β-actin
E
F
G
Flu (μM) 0 0.1 1
H
I

Fig S4

Vehicle

Fluvastatin

Afatinib

Afatinib+
fluvastatin

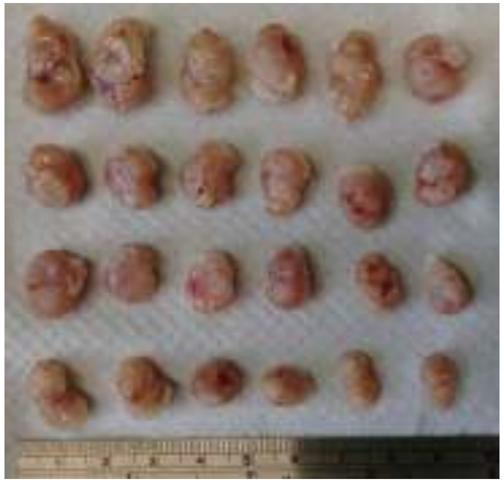

Fig S5

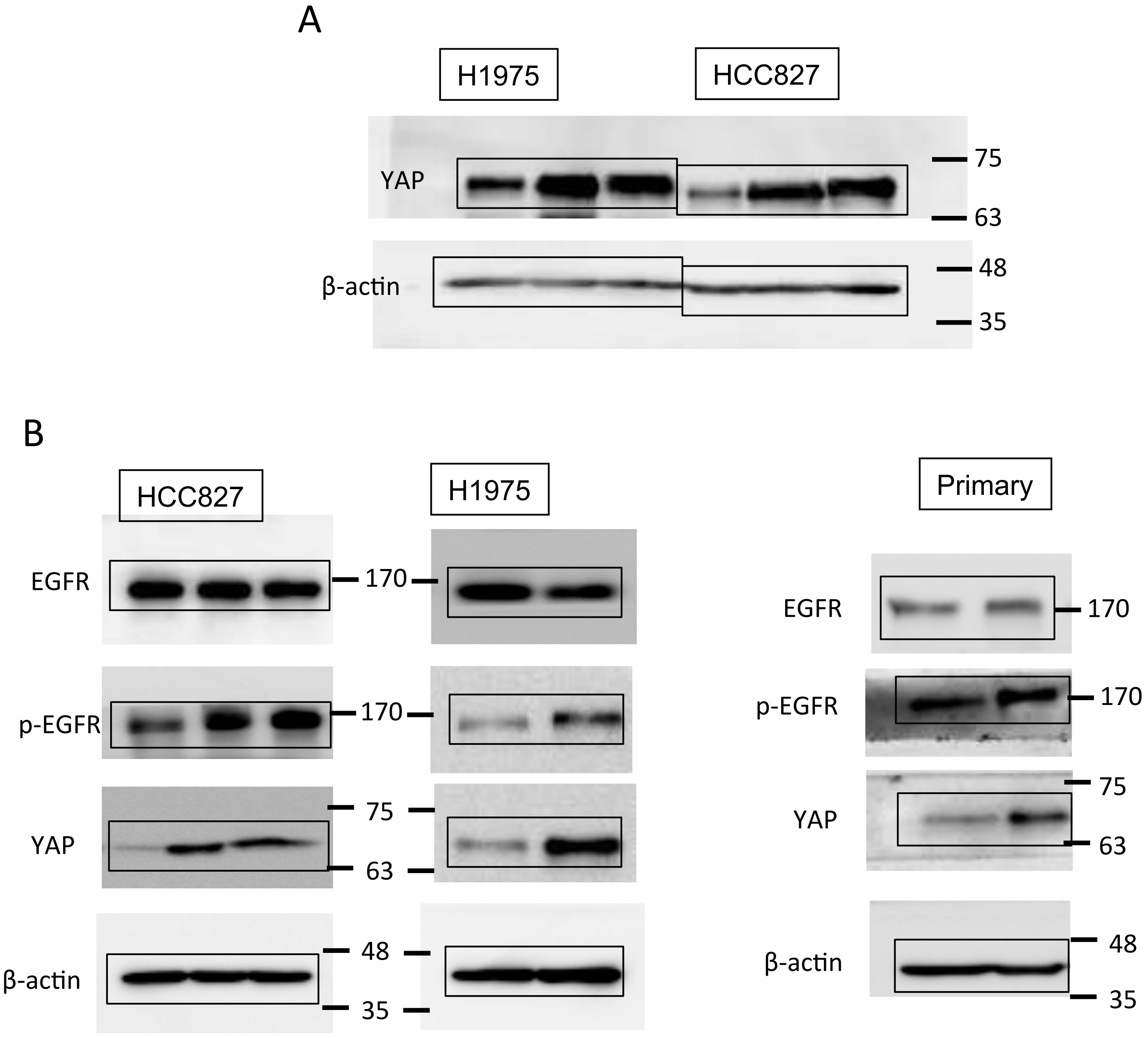
A
H1975
HCC827
YAP
75
63
β-actin
48
35
B
HCC827
H1975
Primary
EGFR
170
p-EGFR
170
YAP
75
63
β-actin
48
35

Fig S6

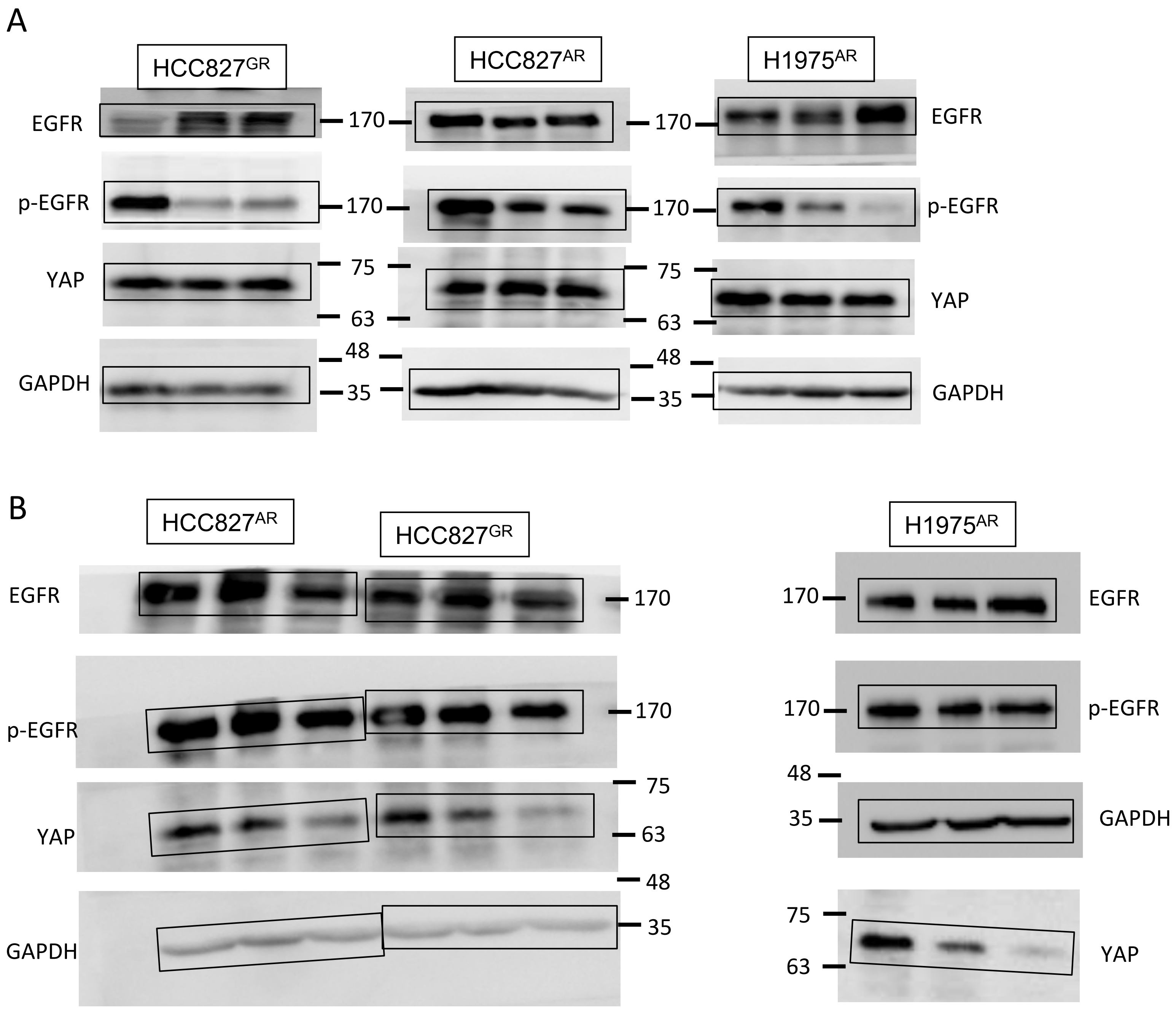
A
HCC827^GR
HCC827^AR
H1975^AR
EGFR
p-EGFR
YAP
GAPDH
170
75
63
48
35
B
HCC827^AR
HCC827^GR
H1975^AR
EGFR
p-EGFR
YAP
GAPDH
170
75
63
48
35

Fig S7

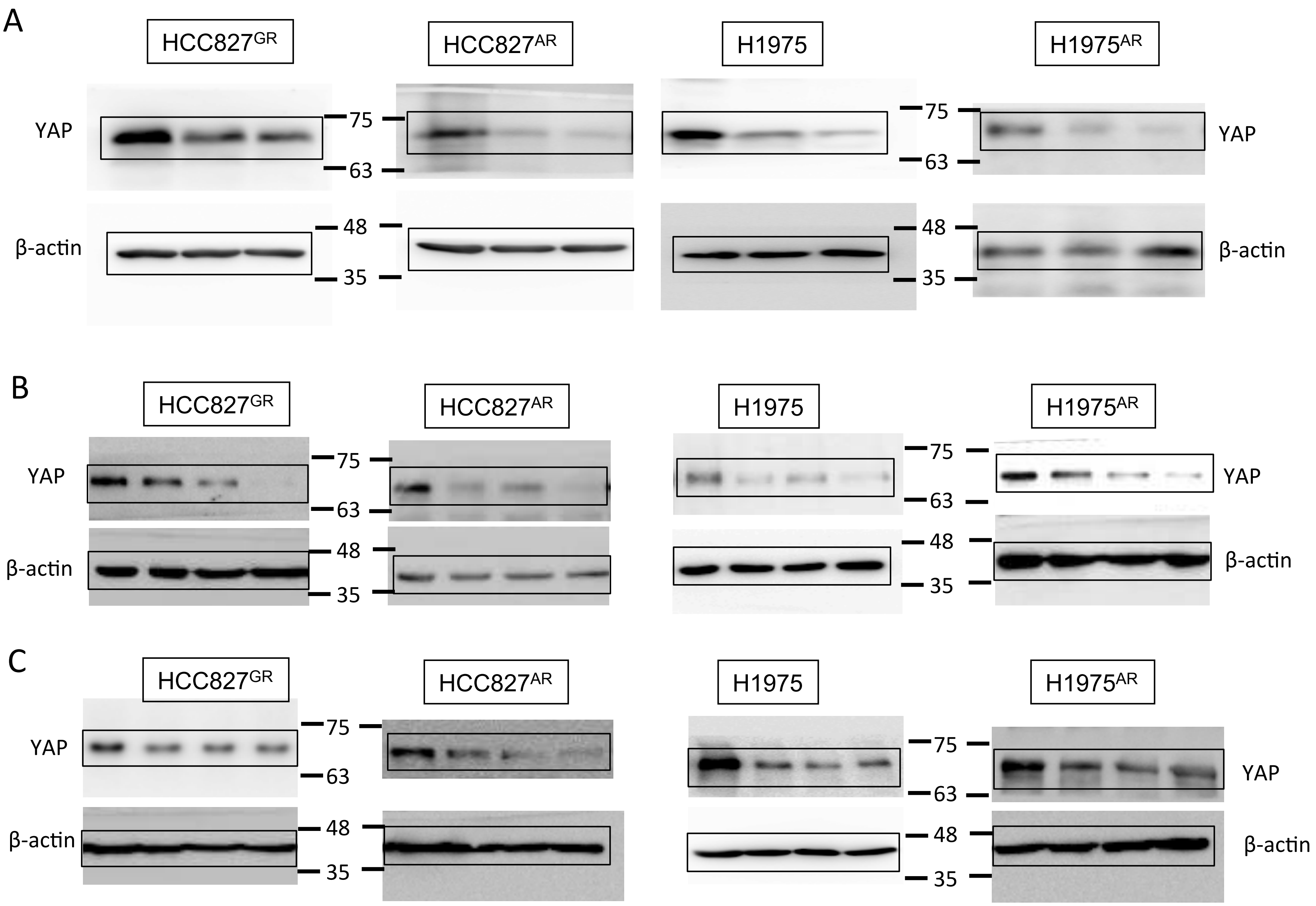
A
HCC827GR
HCC827AR
H1975
H1975AR
YAP
β-actin
75
63
48
35
B
HCC827GR
HCC827AR
H1975
H1975AR
YAP
β-actin
75
63
48
35
C
HCC827GR
HCC827AR
H1975
H1975AR
YAP
β-actin
75
63
48
35

Fig S8

A. Original blots of FigS2

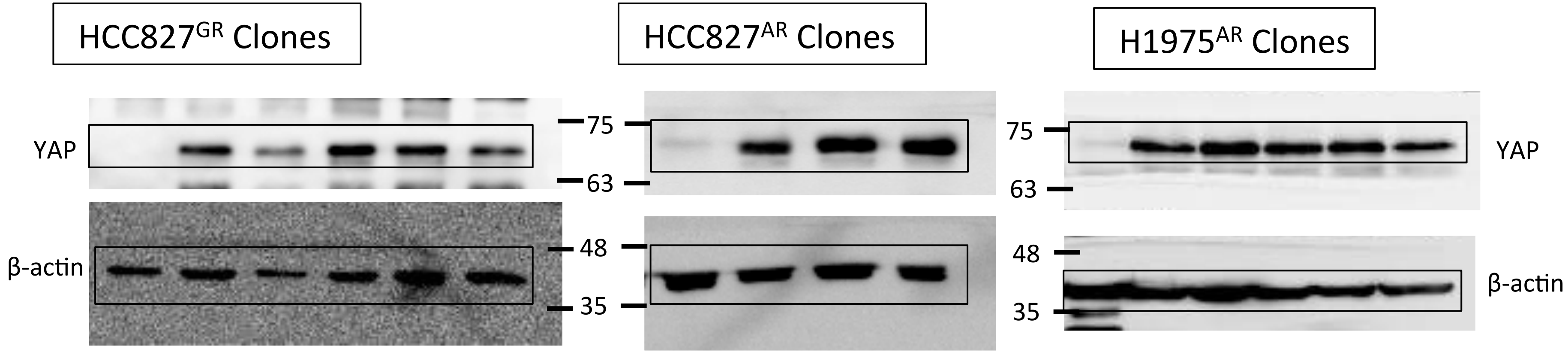

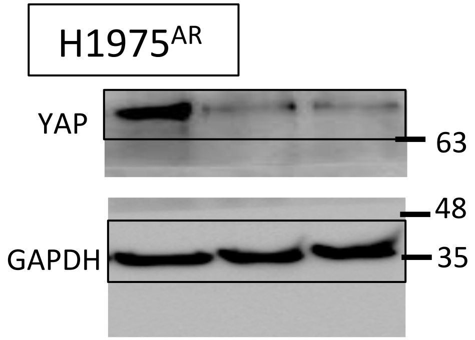

B. Original blots of FigS3

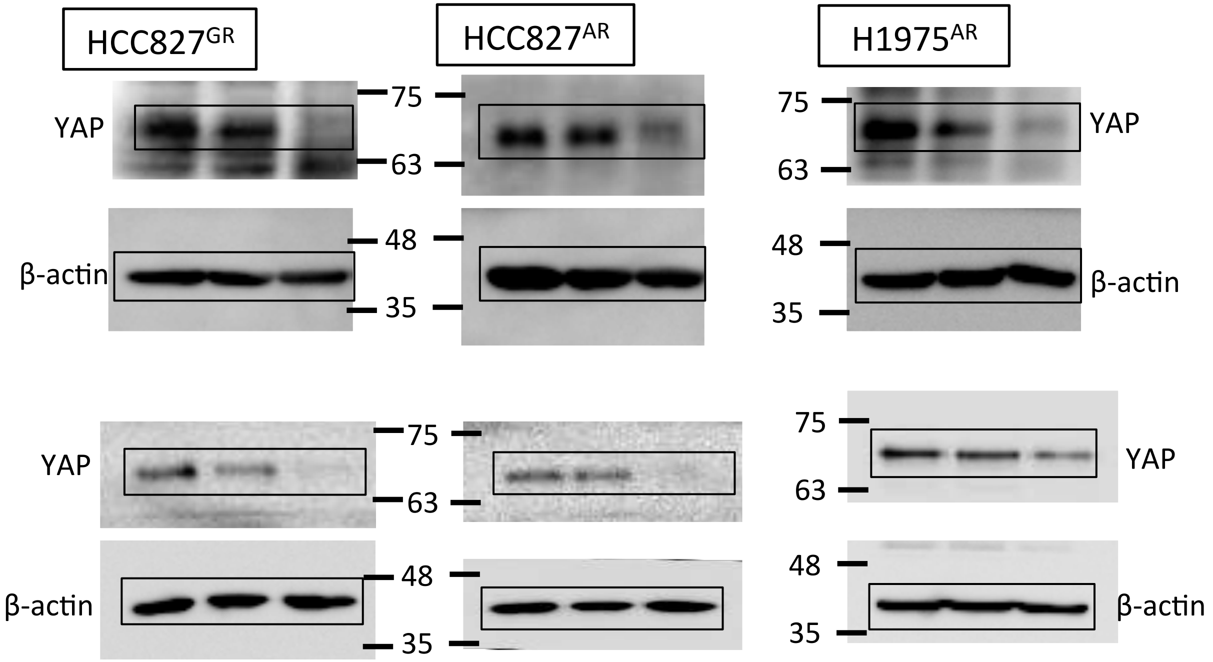

Supplement: Supplementary file 1 — Supplementary Information [file 41598_2017_18527_MOESM1_ESM.pdf]
